# Overexpression of *pink1* or *parkin* in indirect flight muscles promotes mitochondrial proteostasis and extends lifespan in *Drosophila melanogaster*

**DOI:** 10.1371/journal.pone.0225214

**Published:** 2019-11-12

**Authors:** Hongbin Si, Peng Ma, Qiying Liang, Youjie Yin, Ping Wang, Qi Zhang, Saifei Wang, Hansong Deng

**Affiliations:** 1 College of Animal Sciences and Technology, Guangxi University, Nanning, China; 2 Shanghai East Hospital, School of Life Sciences and Technology, Tongji University, Shanghai, China; University of Texas Medical School at Houston, UNITED STATES

## Abstract

Dysfunctional mitochondria have been implicated in aging and age-related disorders such as Parkinson’s diseases (PD). We previously showed that *pink1* and *parkin*, two familial PD genes, function in a linear pathway to maintain mitochondrial integrity and function. Studies of mammalian cell lines also suggest that these genes regulate mitochondrial autophagy(mitophagy). Overexpressing Parkin promotes proteostasis and function of aged muscles both in fruit flies and mice, and recent studies also indicated that mitochondrial ubiquitination are accumulated in aged muscles. However, the underlying mechanisms for pink1 and parkin mediated mitophagy on longevity is not fully understood. Here, we found that mitochondrial ubiquitination increased in indirect flight muscles (IFMs) in an age-dependent manner. Overexpression of *pink1* or *parkin* in IFMs can abolish mitochondrial ubiquitination, restore ATP level and extend lifespan, while blocking autophagy via ATG1 knock-down suppress these effects in aged IFMs. Taken together, these results show that *pink1/parkin* promotes mitophagy of mitochondrial ubiquitination in aged muscles and extend lifespan in an Atg1-dependent manner. Our study provides physiological evidence that mitophagy of mitochondrial ubiquitination mediated by PINK1/ Parkin is crucial for muscle function and highlights the role of mitophagy in the pathogenesis of chronic diseases like PD.

## Introduction

Mitochondrial dysfunction and accumulation of mitochondrial DNA mutations are hallmarks of chronic diseases, including neurodegenerative diseases [1, 2]. Mitochondria are plastic and extremely dynamic organelles. Besides biogenesis, fission-fusion and transportation, mitochondrial autophagy(mitophagy) is proposed to play an important role in mitochondrial quality control[3, 4]. *pink1* (PTEN induced putative kinase 1) and *parkin* are two familial genes associated with Parkinson’s diseases (PD). We and others have shown that *pink1* and *parkin* function in the same genetic pathway to regulate mitochondrial dynamics[5–8]. Recent studies in mammalian cell lines have shown that Pink1 is stabilized in depolarized mitochondria and recruits the E3 ubiquitin ligase Parkin, where it ubiquitylates several target proteins on the outer mitochondrial membrane[9]. Ubiquitinated mitochondria are then degraded by proteasomes and autophagy through p62 (SQSTM1), or NDP52 and optineurin, autophagy cargo receptors that can transport ubiquitylated proteins to autophagosomes via interaction with LC3 [10, 11]. However, PINK1/Parkin mediated mitophagy has largely been elucidated in cell lines treated with uncouplers, such as CCCP. Recent studies in Drosophila showed that ubiquitinated proteins accumulated in aged muscles[12, 13], Parkin overexpression promotes proteostasis and tissue function of aged muscles both in Drosophila and mice [13, 14]. Meanwhile, studies in Drosophila also demonstrated that mitochondria are ubiquitinated in aged muscles, and overexpression of Drp1 or P62 can decrease mitochondrial associated ubiquitination in muscles and extend lifespan [15, 16]. On the other hand, loss of pink1 or parkin in Drosophila block mitophagy in an age-dependent manner[17, 18]. Whether boosting pink1 or parkin can directly regulate mitochondrial ubiquitination and turnover in aged muscles, however, is not fully characterized.

Our recent studies failed to detect Pink1 stabilization or Parkin recruitment in mitochondria in Drosophila indirect flight muscles (IFMs) after acute genetical or pharmacological uncoupling [19]. Similarly, Parkin recruitment was not observed in respiratory chain-deficient mitochondria in mouse dopamine neurons in vivo [20]. We further showed that segregation of damaged mitochondria from the network by increasing mitochondria fission is a prerequisite for subsequent mitophagy[19]. By contrast, promoting mitochondrial fission in midlife was shown to extend healthy lifespan in *Drosophila melanogaster* [15].

Hence, we propose that mitophagy is undetectable in these circumstances is due to strong rejuvenating capacity mediated by mitochondria dynamics in young animals, which declines with age.

To test this hypothesis, we examined the role of PINK1/Parkin mediated mitophagy in aged Drosophila muscles.

## Materials and methods

### Drosophila genetics and strains

UAS-Atg1^6A^ and UAS-Ref(2)PGFP were obtained from Dr. Thomas Neufeld, UAS::Atg1^RNAi^ (BL44034) were obtained from the Bloomington Drosophila Stock Center. IFMGAL4, UAS-pink1, UAS-parkin^C2^ flies have been previously described [21]. Drosophila strains were raised on standard medium at 25°C with 12hr day/night cycle unless otherwise specified. For life span experiments, flies were collected under and housed at a density of 30 male or female flies per vial. Around 100–120 flies for each genotype were scored and all flies were kept in a humidified, temperature-controlled incubator with 12 h on/off light cycle at 25°C. Flies were flipped to fresh vial every 2–3 days and scored for death.

### Generation of UAS-TOM20-mCherry transgenic flies

mCherry has been fused in-frame into the C-terminal end of the endogenous TOM20 open reading frame and sequentially subcloned into pUAST vector with restriction enzyme sites EcoRI/Not1 and Not1/XhoI respectively. Plasmid was sequence verified before injected into W^1118^ following standard germline injection procedure (Rainbow Transgenic Flies, Inc.). Primers used:

mCherry F 5’-ggccGCGGCCGC ATGGTGAGCAAGGGCGAGG-3’

mCherry R 5’- ggccCTCGAG CTTGTACAGCTCGTCCATGCCG-3’

Tom20 F 5’- ggccGAATTC ATGATTGAAATGAACAAAAC-3’

Tom20 R 5’- ggccGCGGCCGC CTATTCGAGGTCGTCGATACT-3’

### ATP measurement

Muscle ATP level was measured using a luciferase-based bioluminescence assay (ATP Bioluminescence Assay Kit HS II, Roche Applied Science). For each measurement, 5 thoraces were freshly dissected and immediately homogenized in 50 μl lysis buffer. The lysate was boiled for 5 min and cleared by centrifugation at 20,000 g for 1 min. 5 μl of cleared lysate was added to 90 μl dilution buffer and 5 μl luciferase, and the luminescence was immediately measured using a 96 well plate luminometer. Each reading was converted to the amount of ATP per thorax based on the standard curve generated with ATP standards. The readings will then be normalized with the protein level measured by BCA Bradford assay. Three independent measurements were made for each genotype.

### Mitochondrial mass quantification

Mitochondria were visualized by fluorescent microscopy with mitochondria targeted mitoGFP or by light microscopy with Toludine Blue staining. Mitochondria from about 20 muscles of 5 thoraxes were analyzed based on the relative intensity and normalized with control.

### Climbing assay

Groups of twenty flies were placed in an empty climbing vial and then tapped down to the bottom. They were allowed 10 seconds to climb past a dotted line marked 5 cm from the bottom of the vial. The number of flies above the 5 cm mark at 10 seconds was recorded as a percentage of flies able to climb/vial. At least 5 separate trials were run per genotype.

### Embedding, sections, and Toluidine blue staining

Thoraces from young female flies were dissected, fixed in paraformaldehyde/glutaraldehyde, post-fixed in osmium tetraoxide, dehydrated in ethanol, and embedded in Epon. After polymerization of Epon, blocks were cut to generate 1.5-μm thick sections using a glass knife on a microtome (Leica, Germany). Toluidine blue was used to stain 1.5-μm—thick tissue sections.

### Immunofluorescence, Immuno-blot and confocal microscopy

For muscles, thoraces were dissected and fixed in 4% paraformaldehyde in phosphate buffered saline (PBS). After thoraces were washed three times in PBS, muscle fibers were isolated and stained with rhodamine phalloidin (Invitrogen, 1:1000) in PBS+1% Triton X-100. For antibody staining, muscle fibers were permeabilized and blocked in PBS+0.1% Triton X-100, and incubated in primary and secondary antibodies diluted in PBS+1% BSA. The following primary antibodies are used: mouse anti-ATP Synthase (Mitosciences, Eugene, OR) and Mouse anti-FK2 (Enzo Life Sciences, Farmingdale, NY). For Westernblot, anti-Tom20 (Y413613, abmgoods, B.C. Canada). All images were taken on a Zeiss LSM5 confocal microscope.

### Lysotracker staining

Indirect flight muscles were freshly dissected in PBS and incubated in dark chamber within PBS contains LysoTracker® Red DND-99 (Life technologies, L-7528, dissolved in 100% ethanol with final concentration 1μM) for 10min. After briefly rinse and wash in PBS, muscles were mounted in PBS and immediately imaged under confocal microscopy.

### Isolation of mitochondria enriched population

The protocol is largely based on previous study with minor modifications [15]. In brief, around 50 thoraces of each condition were gently crushed in chilled mitochondrial isolation buffer [250 mM sucrose, 10 mM Tris-HCl (pH 7.4), 0.15 mM MgCl2] using a plastic pestle homogenizer and then spun twice at 500×g for 5 min at 4°C to remove debris. The supernatant was then spun at 5000×g, for 5 min at 4°C to get the mitochondrial enriched pellets.

## Results

### Age-dependent increase of mitochondria ubiquitylation in Drosophila IFMs

We first examined the status of mitochondrial damage during aging in IFMs. Mitochondria in IFMs align along the muscle fiber, and we previously developed a convenient method for visualizing mitochondria morphology under a confocal microscope[8].

**Fig 1:**
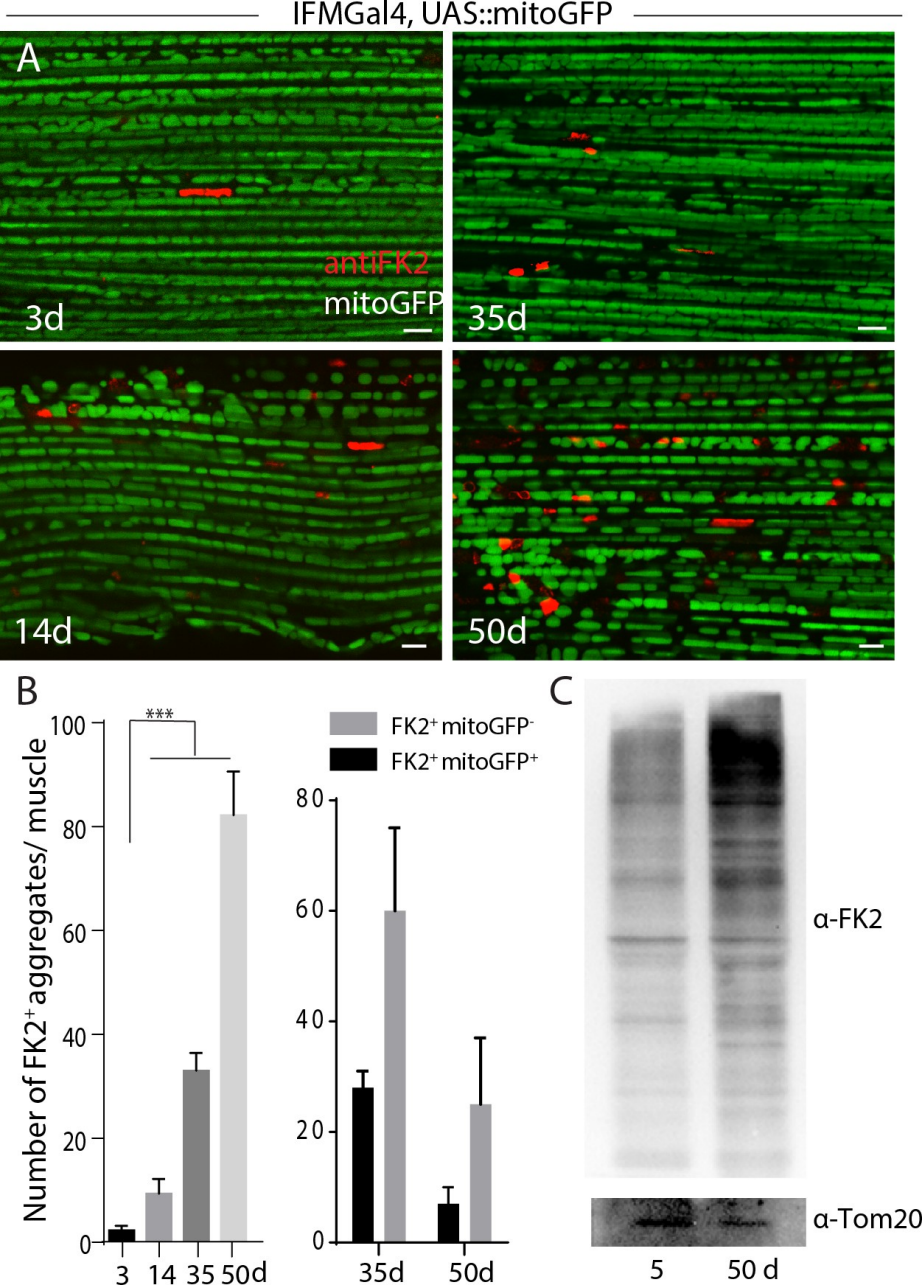
Ubiquitylated mitochondria are progressively accumulated during aging in Drosophila indirect flight muscles. A, FK2-positive ubiquitin conjugates in Drosophila IFMs were examined at different stages. B, Left, quantification of A, around 30 muscles from 5~6 animals of each time point was quantified. One-way ANOVA performed for statistics, and p<0.001 for S.E.M. Right, colocalization of mitoGFP with FK2 positive foci was quantified in muscles from 35d or 50d-old animals. Scale bar: 5 μm. C, Western-blot staining against FK2 in mitochondrial sub-fractionation of thoraces from different ages. Genotype: IFMGal4;UAS-Ref(2)PGFP.

Ubiquitination of the mitochondrial outer membrane protein is a prerequisite for autophagosome recruitment via adaptor proteins [22]. We sought to test whether mitochondria were ubiquitylated in IFMs. FK2 is an antibody against mono and poly-ubiquitinylated protein conjugates. As shown in **Fig 1A,** few FK2 positive foci were present in the muscles of young flies (3–4 days old), while size and number were significantly higher in the muscles of 14-day old flies and reached 2–5 μm in muscles of 35-day-old flies **(Fig 1A and 1B)**. Notably, a significant population of FK2-positive puncta are located along the muscle fibers, and their shape resembles mitochondria. To test whether a subset of these FK2-positive foci was mitochondria, a mitochondria matrix targeted GFP was expressed in muscles under the control of the UAS-GAL4 system by IFMGal4, which is exclusively expressed in indirect flight muscles in late pupae and adult stage [21]. As shown in **S1 Fig**, FK2 positive puncta are colocalized or intercalated with mitoGFP, suggesting a subset of mitochondria were ubiquitylated in muscles. Furthermore, isolated mitochondria of throaces from aged flies (50 days old) have significant more FK2 signals compared with those in young flies (5 days old) (**Fig 1C**).

**Fig 2:**
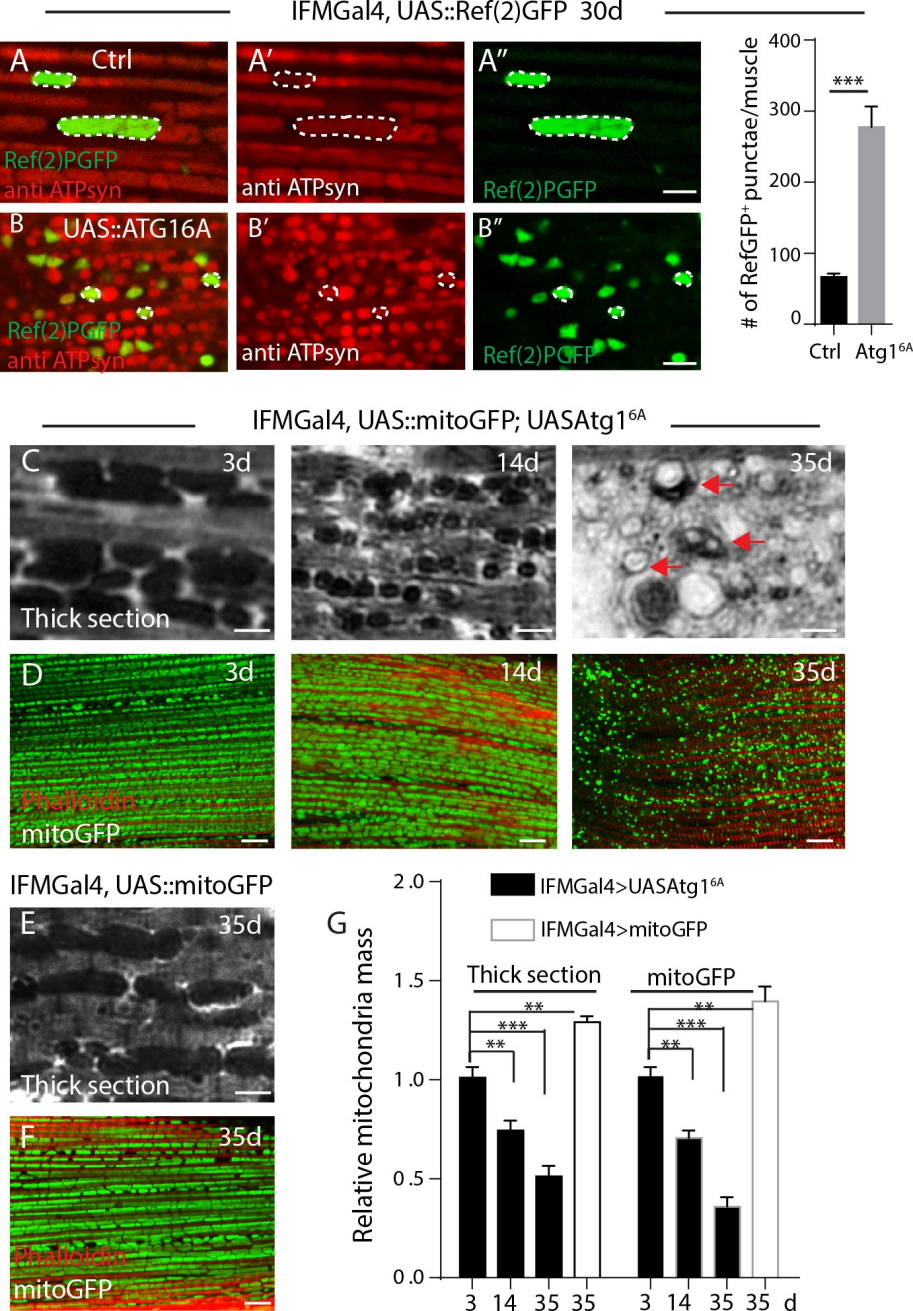
Age dependent accumulation of ubiquitinated mitochondria proteins are recycled by Atg1-related autophagy. A-B, IFMs of 30-day old flies expressing Ref(2)PGFP (in green) was labeled with anti-ATP syn(red) for mitochondria. Typical mitochondria are circled in dashed lines. Genotypes: A, IFMGal4; UAS::Ref(2)PGFP, and B, IFMGal4; UASAtg1^6A^, UAS::Ref(2)PGFP. C, Thick section of muscles from different time points, “vacuolation” of mitochondria are denoted as arrows. Mitochondria are densely stained by Toluidine blue in C and E. D, Mitochondrial mass indicated by matrix targeted mitoGFP (green) from different time points. Muscle fibers were stained with Phalloidin in red. Genotype in C-D, IFMGal4; UAS::mitoGFP, UASAtg1^6A^. E, Thick section of muscles from 35-d old control flies. F, Mitochondrial mass indicated by matrix targeted mitoGFP (green) in control flies, and Phalloidin stained muscle fibers in red. Genotype in E and F: IFMGal4; UAS::mitoGFP. G, Relative mitochondria mass in C-F was quantified. At least 20 muscles from 6 thoraxes of each condition were analyzed. t-Test was performed for statistics, p value for S.E.M, **: p<0.01, ***: p<0.001.

To examine the fate of these FK2 positive mitochondria, we introduced Ref(2)P, the Drosophila homolog of P62 [23], which can selectively target ubiquitinated proteins for autophagical degradation [24]. Previous studies have shown that Ref(2)P localizes to protein aggregates in Drosophila aged brain [23]. As shown in **S2 Fig**, Ref(2)PGFP-positive puncta in aged IFMs tightly colocalize with FK2 staining in IFMGal4>Ref(2)GFP flies, suggesting that Ref2P binds with ubiquitin in IFMs as well. Furthermore, Ref(2)PGFP-positive puncta were substantially co-stained with mitochondrial markers, such as an antibody against ETC complex V subunit ATP Synthase **(Fig 2A)**, and a mitochondrial outer membrane targeted mCherry, in aged IFMGal4>Ref(2)PGFP muscles (**S3 Fig**).

Together, these results indicated that mitochondria in indirect flight muscles are progressively ubiquitylated during aging, which is consistent with previous studies [15].

### Autophagy promotes mitochondrial turnover in aged IFMs

We then examined the fate of these mitochondrial ubiquitination. ATG1/ULK1 is crucial for autophagy initiation[25]. As shown in **Fig 2A and 2B**, overexpression of Atg1 renders more Ref(2)PGFP positive mitochondria present in aged IFMs than controls. Meanwhile, mitochondria mass, indicated by densely dark signals in thick sections of IFMs with Toluidine blue staining[26] **(Fig 2C)** and mitoGFP **(Fig 2D)**, are progressively decreased in Atg1 OE flies compared with age-matched controls **(Fig 2E–2G).**

These results demonstrated that an augmented Atg1 level is sufficient to induce mitochondrial ubiquitination and subsequent clearance.

### Overexpression of *pink1* or *parkin* in IFMs suppresses mitochondrial ubiquitination, restores ATP level and muscle function

**Fig 3:**
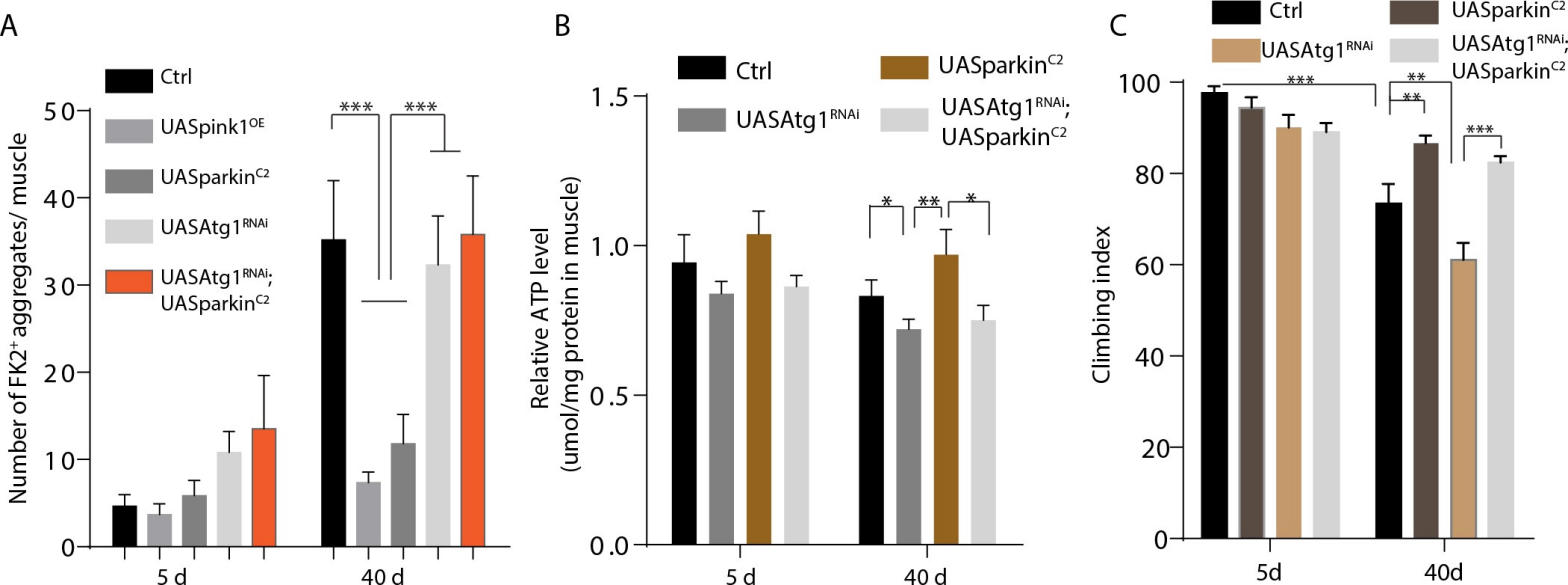
Overexpression of *pink1* or *parkin* in IFMs promotes mitochondrial proteostasis and muscle function in an Atg1 dependent manner. A, Quantifications of FK2-positive aggregates per muscle of different genotypes at different time points. At least 20 muscles from 6 animals were quantified. One-Way ANOVA analysis performed for statistics, p value for S.E.M, ***: p<0.001. B, Quantification of relative ATP level from muscles with different genotypes. ATP level was normalized with protein level from six thoraces, and triplicates was tested for each genotype. t-Test was performed for statistics, p value for S.E.M, *: p<0.05, **: p<0.01. C, Climbing ability was examined. 20 flies of each genotype at each condition was measured, five independent repeats were run for each condition. t-Test was performed for statistics, p value for S.E.M, **: p<0.01, ***: p<0.001. Genotypes for A-C, IFMGal4>UASpink1^OE^, IFMGal4>UASparkin^C2^, IFMGal4>UASAtg1^RNAi^, IFMGal4>UASAtg1^RNAi^, UASpark^C2^.

We sought to test whether Pink1 or Parkin level was responsible for ubiquitination and quality control of mitochondria in aged muscles in Drosophila. Pink1 or Parkin were genetically overexpressed in IFMs by IFMGal4. As shown in **Fig 3A**, FK2-positive puncta were significantly reduced in 35-day old muscles of Pink1 overexpressing (Pink1 OE) or Parkin overexpressing (Parkin^C2^) flies. Cellular ATP is mainly produced in mitochondria by oxidative phosphorylation. As shown in **Fig 3B and S4 Fig**, the ATP level in muscle fiber was also significantly restored in Pink1 OE or Parkin^C2^ flies. As an indicator of muscle function, climbing ability reduced in aging animals was significantly restored by overexpressing Pink1 or Parkin in muscles (**Fig 3C and S4 Fig**).

### Overexpression of *pink1* or *parkin* in IFMs extends lifespan in an autophagy dependent manner

**Fig 4:**
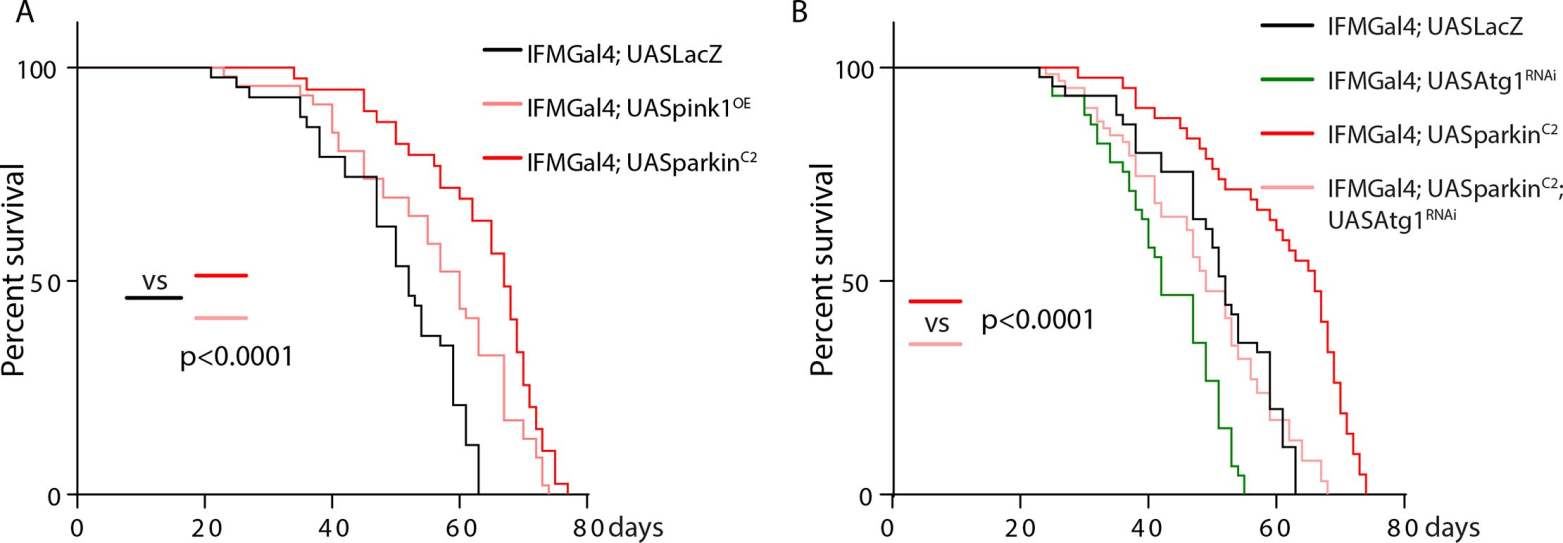
Overexpression of *pink1* or *parkin* in IFMs extends lifespan in an Atg1 dependent manner. A-B, Survival rate was analyzed for different genotypes. Around 100–120 flies for each genotype was examined. Log-rank (Mantel-Cox) test was run for statistics.

Since mitochondrial ubiquitination is accompanied with aging. We then test whether the longevity is regulated by pink1/parkin. Indeed, lifespan was significantly extended in IFMGal4; UASPink1 (median lifespan: 70.8 days, around 7.5% increase) and IFMGal4; UASParkin^C2^ flies (median:73.5 days, around 11% increase) than controls (median: 65.1 days) **(Fig 4A).** We next sought to test whether autophagy is responsible for clearance of ubiquitylated mitochondria downstream of pink1 and parkin. A rate-limiting step of mitophagy is degradation of mitochondria in the autolysosome by acidic lysosomes [4]. Acidic cellular compartments, including lysosomes can be stained by Lysotracker. As shown in **S5 Fig.**, aged muscles in control animals contain aberrant enlarged mitoGFP positive organelle which are negative for Lysotracker, while more Lysotracker positive vesicles shows colocalized with mitoGFP in Parkin^C2^ counterparts. These results indicated Parkin overexpression promotes mitophagy in aged muscles.

Our previous results indicated that knocking down Atg1 in IFMs blocks autophagy, while overexpressing Atg1 promotes both mitochondrial fission and autophagy [19]. As shown in **Fig 4B**, *Atg1* knock-down substantially block the rescuing effect of Parkin overexpression in aged IFMs in terms of mitochondrial ubiquitylation, ATP level, climbing ability and lifespan (**Figs 3 and 4B**).

Taken together, these results indicate that *Atg1* and autophagy are downstream of *pink1* and *parkin* to maintain the mitochondrial proteostasis in Drosophila IFMs.

## Discussion

Mitophagy is considered as an essential process to maintain mitochondrial quality [27]. The PINK1/Parkin) pathway is among the best characterized pathways through which mitophagy is induced in mammalian cells[28]. However, the majority of studies have utilized acute mitochondrial uncouplers, such as CCCP, which are difficult to recapitulate in vivo. Studies have shown that Pink1 overexpression is beneficial for certain neurodegenerative models, such as Huntington’s Disease [29] and α-synuclein model in Drosophila [30]. Meanwhile, Parkin overexpression was shown to decrease ubiquitinated proteins accumulated in aged muscles and promote lifespan[13, 15]. Whether these pink1/parkin mediated beneficial effects depend on mitophagy, however, is less clear. In fact, we did not observe any mitophagy in Drosophila IFMs by acute mitochondrial insults[19]. On the other hand, studies have shown that mitophagy increases with aging, and this age-dependent increase is abolished by loss of Pink1 or parkin in Drosophila[17, 18]. Mitophagy is a terminal process to recycle severely damaged mitochondria. Mitochondrial homeostasis is maintained by the balance between rejuvenating and damaging events. Dysfunctional mitochondria accumulate in aged animals, suggesting that the capacity to rejuvenate is overtaken by mitochondrial damage. Rana et al. (2017) have shown that a subset of mitochondria are ubiquitinated in aged muscles [15], whether mitochondrial ubiquitination can be abolished by *pink1/parkin overexpression* is not well characterized.

Here, we found that mitochondrial associated ubiquitination is accumulated in aged muscles, and increasing Pink1 or Parkin level in indirect flight muscles was sufficient to promote mitochondrial quality and extend lifespan in an autophagy-dependent manner.

These results indicate that accumulation of ubiquitylated mitochondria is a hallmark of muscle aging in Drosophila and that *pink1/parkin* can promote mitochondrial proteostasis, facilitate clearance of damaged mitochondria, and extend lifespan in Drosophila.

## Supporting information

S1 Fig FK2 positive aggregates are colocalized or intercalated with mitoGFP in Drosophila IFMs. (DOCX)

S2 Fig FK2 positive aggregates are colocalized with Ref(2)PGFP in Drosophila IFMs. (DOCX)

S3 Fig Characterization of a mitochondrial outer membrane targeted genetic reporter. (DOCX)

S4 Fig Overexpression of *pink1* in IFMs promotes muscle function in an Atg1 dependent manner. (DOCX)

S5 Fig Parkin overexpression promotes mitochondrial degradation by acidic lysosomes in aged muscles. (DOCX)
