# Supplementary material for: Overexpression of pink1 or parkin in indirect flight muscles promotes mitochondrial proteostasis and extends lifespan in Drosophila melanogaster
Source: PLoS One. 2019 Nov 12;14(11):e0225214. doi: 10.1371/journal.pone.0225214 (PMC6850535; doi:10.1371/journal.pone.0225214)
Supplement: S1 Fig — (DOCX) [file pone.0225214.s001.docx]

**Supplemental Figure 1** **FK2 positive aggregates are colocalized or intercalated with mitoGFP in Drosophila IFMs**

A, Ubiquitin positive conjugates was stained by FK2 in IFMs of IFMGal4, UASmitoGFP flies.

A’-A’’’ are high magnification of boxed area of A. Scale bars are 2μm. **
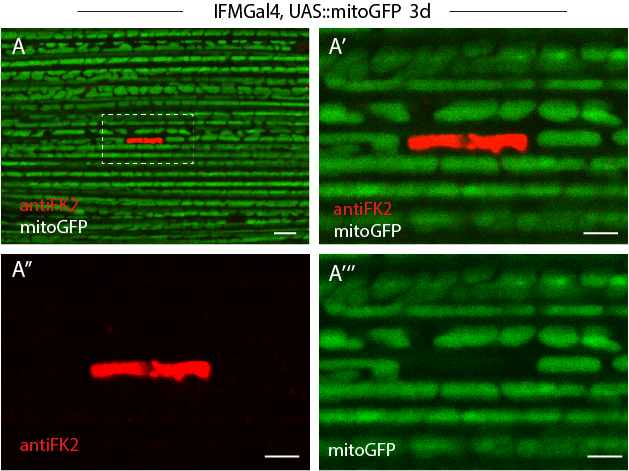
**
