# Supplementary material for: Overexpression of pink1 or parkin in indirect flight muscles promotes mitochondrial proteostasis and extends lifespan in Drosophila melanogaster
Source: PLoS One. 2019 Nov 12;14(11):e0225214. doi: 10.1371/journal.pone.0225214 (PMC6850535; doi:10.1371/journal.pone.0225214)

**Supplemental Figure 3 Characterization of a mitochondrial outer membrane targeted genetic reporter**

A, C-terminal of TOM20 was in-frame fused with mCherry under the control of UAS and co-expressed with mitochondrial matrix targeted UASmitoGFP by IFMGal4. A’-A’’’ are high mag pictures of boxed area of A. scale bars are 2μm.

B, Ref(2)PGFP and mitochondrial outer membrane targeted mCherry was expressed in IFMs by IFMGal4. B’-B’’’ are higher mag images of boxed area in D. Scale bars are 2μm.


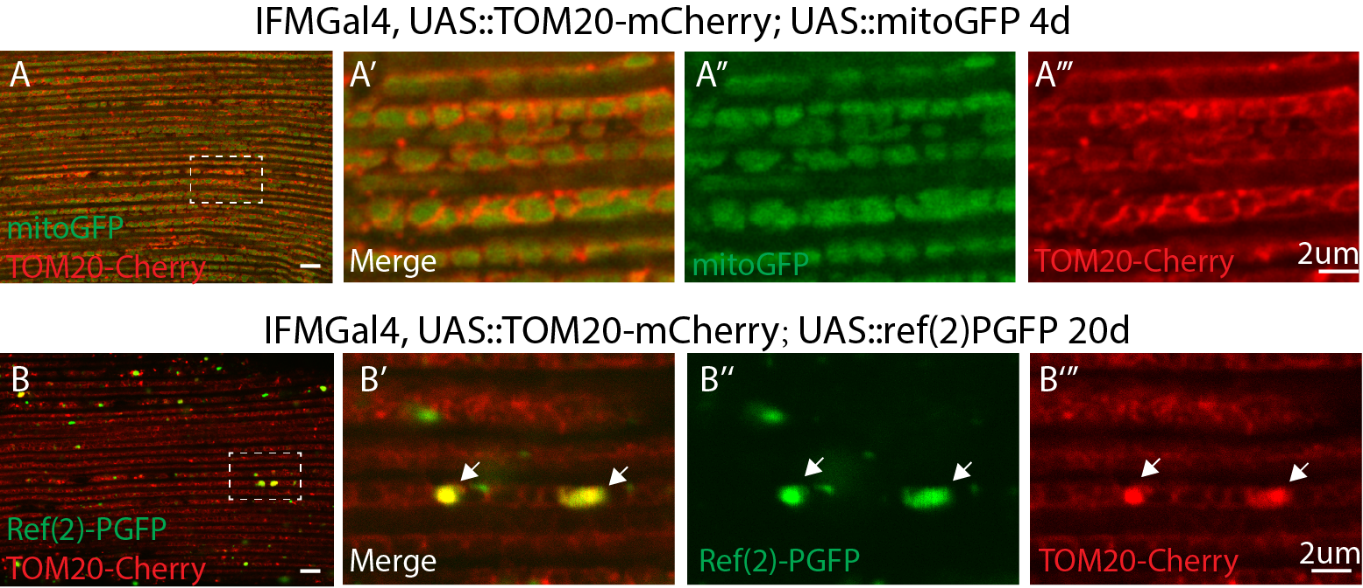

Supplement: S3 Fig — (DOCX) [file pone.0225214.s003.docx]
