# Supplementary material for: Overexpression of pink1 or parkin in indirect flight muscles promotes mitochondrial proteostasis and extends lifespan in Drosophila melanogaster
Source: PLoS One. 2019 Nov 12;14(11):e0225214. doi: 10.1371/journal.pone.0225214 (PMC6850535; doi:10.1371/journal.pone.0225214)

**Supplemental Figure 4 Overexpression of *pink1* in IFMs promotes muscle function in an Atg1 dependent manner.**

A, Quantification of relative ATP level from muscles with different genotypes. ATP level was normalized with protein level from six thoraces, and triplicates was tested for each genotype. t-Test was performed for statistics, p value for S.E.M, *: p<0.05, **: p<0.01.

B, Climbing ability was examined. 20 flies of each genotype at each condition was measured, five independent repeats were run for each condition. t-Test was performed for statistics, p value for S.E.M, **: p<0.01, ***: p<0.001.


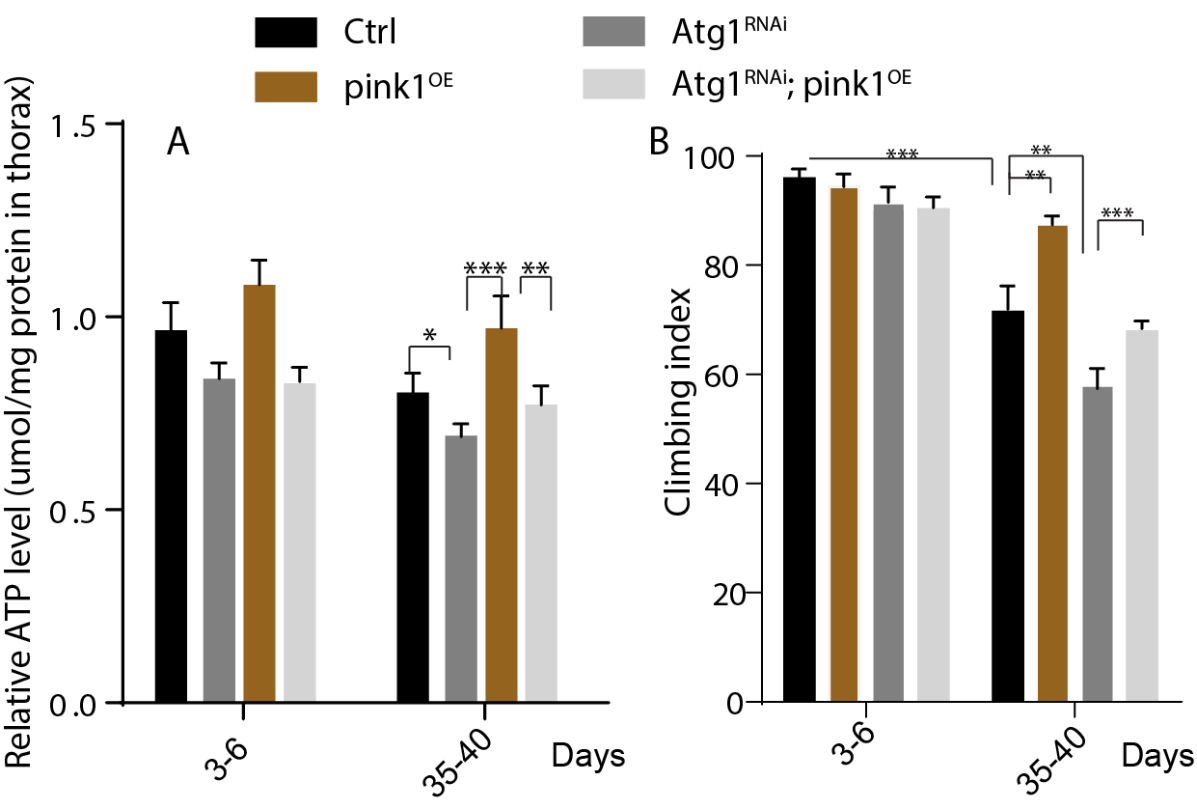

Supplement: S4 Fig — (DOCX) [file pone.0225214.s004.docx]
