# Supplementary material for: Overexpression of pink1 or parkin in indirect flight muscles promotes mitochondrial proteostasis and extends lifespan in Drosophila melanogaster
Source: PLoS One. 2019 Nov 12;14(11):e0225214. doi: 10.1371/journal.pone.0225214 (PMC6850535; doi:10.1371/journal.pone.0225214)

**Supplemental Figure 5 Parkin overexpression promotes mitochondrial degradation by acidic lysosomes in aged muscles.**

A-D. Fresh dissected muscles from animals with indicated genotypes were incubated with Lysotracker, a dye for acidic lysosomes. Mitochondria was labeled by mitoGFP. Abnormal enlarged mitochondria in control aged muscles were denoted in arrow. Scale bar: 10um.

E. Colocalization of mitoGFP with Lysotracker positive (Lyso+) vesicles was quantified. t-Test was performed for statistical analysis. **: p<0.01, S.E.M was shown. Around 60-80 muscles from 10 animals of each genotype was analyzed.


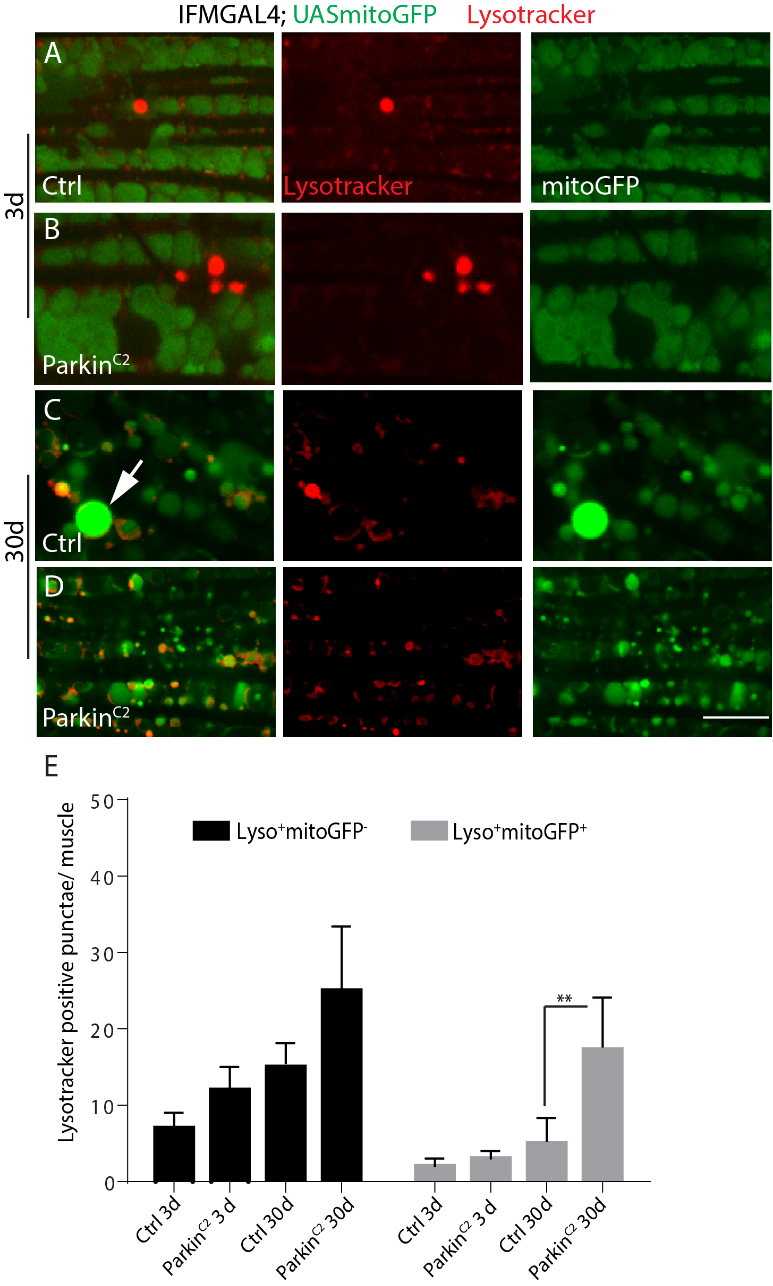

Supplement: S5 Fig — (DOCX) [file pone.0225214.s005.docx]
